# Consumer Behaviour and Attitude towards the Purchase of Organic Products in Riobamba, Ecuador

**DOI:** 10.3390/foods11182849

**Published:** 2022-09-14

**Authors:** Víctor Dante Ayaviri-Nina, Nataly Sthefania Jaramillo-Quinzo, Gabith Miriam Quispe-Fernández, Ilias Mahmud, Ibrahim Alasqah, Talal Ali F Alharbi, Nada Alqarawi, Conrado Carrascosa, Ariana Saraiva, Hani A. Alfheeaid, António Raposo

**Affiliations:** 1 Centro de Investigación Para la Innovación y Desarrollo Regional (CIIDER), Facultad de Ciencias Políticas y Administrativas, Universidad Nacional de Chimborazo (UNACH), Riobamba 060103, Ecuador; 2 Department of Public Health, College of Public Health and Health Informatics, Qassim University, Al Bukairiyah 52741, Saudi Arabia; 3 Department of Psychiatric and Mental Health and Community Health Nursing, College of Nursing, Qassim University, Buraydah 51452, Saudi Arabia; 4 Department of Basic Medical Sciences, Unaizah College of Medicine and Medical Sciences, Qassim University, Unaizah 56432, Saudi Arabia; 5 Department of Animal Pathology and Production, Bromatology and Food Technology, Faculty of Veterinary, Universidad de Las Palmas de Gran Canaria, Trasmontaña s/n, 35413 Arucas, Spain; 6 Department of Food Science and Human Nutrition, College of Agriculture and Veterinary Medicine, Qassim University, Buraydah 51452, Saudi Arabia; 7 CBIOS (Research Center for Biosciences and Health Technologies), Universidade Lusófona de Humanidades e Tecnologias, Campo Grande 376, 1749-024 Lisboa, Portugal

**Keywords:** behaviour, consumer attitude, organic products, market

## Abstract

The development of organic agriculture has been promoted worldwide to improve the nutritional health of families, and Ecuador is no exception. The aim of this research was to identify the behaviour and attitudes of consumers toward the commercialization of organic products in the city of Riobamba. This will help us to understand in what situations the consumers access these foods, the producer position in relation to the market, and to know their attitude towards these products. The study used a quantitative approach, and is descriptive, incorporating the deductive method and a non-experimental design. The techniques used were a survey as a source of data collection, with a sample including 195 consumers. Linear regression was applied to test the hypotheses; this made it possible to identify those demographic and cultural factors that determine, to a lesser extent, consumer purchase behaviour in regard to organic products. The study determined that motivation, emotions, and feelings are significantly related to the consumer’s attitude and purchase of organic products.

## 1. Introduction

The behaviour and attitude of the consumers have an important influence on purchasing processes, as they allow for the establishment of relationships between producers and consumers. Consumers make purchasing decisions based on products’ characteristics, presentation, design, and quality, among other factors [1,2,3,4,5]. They are not only interested in the product and its packaging, but also in the production process and the raw materials and supplies used [6]. The study of consumer behaviour is complex, since several internal and external variables influence it difficult [7]. Attitude affects the choice of a product and explains behavior based on the conduct and beliefs of the consumers [8,9]. Attitude, therefore, exerts influence on purchasing decisions, being the most important factor involved in purchasing processes and consuming products [10].

Consumers opt for a more natural or green choice when they desire for less-harmful products, and there is a preference for healthy foods which results in a good attitude in the consumers [11,12]. When making the purchase of a food product the consumers consider the origin and the brand, as these are important for decision making, and when viewing a little-known product, their attitude towards it will be one of rejection or doubt [13]. Likewise, the consumer will be willing to pay more for products of known healthy origin, such as organic products; that is, their purchase intentions and positive attitudes increase [14,15].

Consumer behaviour regarding the consumption of organic products can be defined by various factors, such as belief in the benefits that they have for health, meaning the nutritional value, certification, taste, quality, and price, in addition to belief in the environmental benefits they can provide; however, these purchase factors are not always enough. Meanwhile, the consumers’ attitude toward agricultural products is changing as the need to guarantee food safety arises [16,17,18]. What is sought is an understanding of the existing dynamics in the production processes and the improvement of technologies in this sector that offer products with added consumer benefits, allowing them to purchase a variety of products. Not knowing the origin and quality of the products could generate mistrust, and consequently a rejection of organic products [19,20,21]. For example, the attitude of consumers towards the purchase of foods of animal origin is based on three dimensions: cognitive, affective, and behavioural [22]. It is striking that some cultures have had organic farming practices for several centuries [23,24,25]. Ecuador is not the exception in terms of the development of organic agriculture. The first projects were begun in the ‘90s, and since then, there has been significant growth in the production of organic products [26,27]. Since then, the production of organic food has experienced in the last ten years, a significant increase in consumption by the population [28].

The commercialization of organic products in Ecuador has a certification system that responds to the sociocultural and economic reality of the localities. The farmers commercialize their products in the markets and free fairs, since they can have direct communication with the consumers and can therefore establish trust relationships [29,30] and it allows coordination of purchase and sale between the producers and consumers [31]. This production and commercialization system is based mainly on the access and consumption of healthy products, where the harmful effects of these foods are very low to ensure a healthy diet for the population [32,33]. Therefore, strengthening and promoting the purchase and consumption of organic products is a challenge, since marketing efforts to highlight their virtues are scarce [34,35]. In relation to this, the correspondence between organic products’ production and legal policies is still incipient, since legally the planting and harvesting processes are protected with greater emphasis on industrialization and not on the more-traditional processes that benefit organic family farming and their markets [36,37]; In other words, organic producers need to offer their products in closed markets or with certain limitations, and not all consumers are aware of the existence of these markets and products [38,39], and therefore, very little is known about the behaviour and attitude of consumers towards these products.

Consumer behaviour and attitude of Ecuadorian households in relation to organic product purchasing have not been addressed from this perspective; not everyone has a consumer culture about the products, either due to lack of knowledge about them or because of high prices, and those who consume them are linked to the search for good health, quality, and conservation of the environment [40,41,42]. Specifically, in relation to attitude as a variable, this has not been addressed in the context of the purchase of organic products in Ecuador. Consequently, this research aimed to learn the behavior and attitude of the consumers in relation to organic products in the city of Riobamba and to determine how behavior and attitude influence the purchase of these products. This will help us to understand in what situations the consumer accesses these products. On the other hand, we aim to determine why they prefer these products or why they do not consume them. Differentiating the attitude from the behavior will allow for an analysis of the complexity of the consumers; that is, does attitude determine the consumption of organic products, or it is the behavior that precedes the attitude? This phenomenon has not been addressed in scientific research. Addressing both variables and highlighting their importance to the scientific field has considerable value, since it covers a field that has been little studied.

## 2. Theoretical Aspects

### 2.1. Consumer Behaviour

The study of consumer behavior is complex, as there are various internal and external variables that influence behavior, and it changes according to the life cycle of the product—from its introduction to the market until its disappearance [43,44,45]. Understanding the benefit or value of the product as perceived by the consumers is one of the strategies that helps to satisfy the needs of consumers [46,47]. Understanding the consumers’ perception of the quality of the product, which influences the attitude of consumers toward purchase is also important [48,49]. Consumer behavior contemplates various elements and actions based on preferences, and stable components are considered in the purchase decisions of consumers [50,51,52]. Not all consumers perceive all product attributes in the same way, so perception is one of the factors that determines their attitude and behavior [53,54]. Therefore, the factors that affect consumer behavior are related to the psychological component, and this implies something more important: knowing the degree of happiness of the consumer [55]; internal factors such as personality, lifestyle, learning, motivations, needs, and impulses; and external factors such as culture, social strata, demographic, and reference groups that would influence consumer behavior [56,57]. In the field of organic products, consumer behavior is related to various factors, such as belief in the benefits they have for health [58], in reference to the nutritional value that products can provide, and labeling, certification, taste, quality, and price can also be considered as key characteristics that consumers take into account, as well as beliefs in the environmental benefits that they can provide [59].

### 2.2. Consumer Attitude

The behaviour of the consumer is related to his or her cognitive and affective attitude towards the product [60]. When the attitude of consumers is positive towards a product, they will recommend its consumption; on the contrary, if their attitude is negative, they will refrain from consuming the product [61]. The attitude of the buyers is based on perceptions that will define the product purchase process and has a psychological tendency that affects the choice of an organic product [62], during which the behavior changes according to the psychological and emotional state of the consumer. A product maybe valued favourably or unfavourably based on the stereotypes held by the consumers. The factors that affect the consumer’s attitude are related to emotions, thoughts, personal relationships, and their feelings, among other things. The most prominent factors are emotions, motivation, and feelings of the consumers [63].

### 2.3. Emotions, Motivation, Feelings

Consumers express emotions when they interact with a product which directly influence their purchasing attitude. Emotions modify the cognitive state of people, where feelings experience positive impulses toward the purchase of a product [64]. One way to stimulate consumer purchase is through positive emotions provoked by a commercial, promotion, or publicity. Meanwhile, motivation is a combination of the actions behind the purchase behaviour [57]. There are approaches that allow it to be determined as the relational vision that focuses on the interactions of organic producers with the market [62,64].

When making a purchase, motivation can be divided into categories, such as altruism, which deals with environmental protection, animal welfare, and productive development; and selfishness, which includes nutritional values and negative opinion on organic food [57]. Consumers can be motivated through highly convincing messages linked to food safety that provoke positive thoughts towards the products, which in turn lead them to obtain more and more arguments in favour of the purchase. Likewise, feeling is a sensory component of a cognitive, complex, and lasting structured experience that has affective dispositions and determines the attitude of the consumers who experiences it; that is, they are dispositions of emotional experiences that have implications towards the purchase of the product, and so it can be said that feelings have characteristics similar to emotions [49]. In addition, consumers’ feelings about an organic product are associated with perceived threats, risks, and rewards or benefits, and motivations are connected to the presentation and impression of the organic products to the consumer [65].

### 2.4. Organic Products

It is important to boost the production of organic products, and the initiative seeks to replace traditional systems with natural processes. Its principles depend on the reality of each region, and producers need access to land, credit, government support, and local markets for the marketing of production [23]. For this reason, organic products are born as an alternative to conventional products. The main characteristics of the organic products are using local resources; not using any chemical products and supporting soil conservation, respecting the environment, and promotion of population health in harmony with the nature [49].

In recent years the production and consumption of organic products have also improved, especially for those who have a healthy lifestyle and promote the consumption of nutritious products distinguished by organic labels and affordable prices [66,67]. Additionally, price and promotion have the greatest impact on consumer acceptance and purchase decisions [68,69]. When the benefits of organic food products for the consumers are perceived favourably and when their desire to buy intersects with their personal and social issues, the consumers demonstrate higher intention to purchase organic food products [70]. The literature indicates that income and price are the central elements to do with consumption; in the field of ecological products, as the income of consumers increases, they will be able to acquire products with higher quality. The consumption of ecological products in the market is recent; therefore, these two components play an important role in purchase and consumption decisions [71,72]. 

Consequently, organic production is based on good practices, encourages the consumption of healthy foods, and promotes a better quality of life [53]. Additionally, the organic food market has actors in the role of both producers and consumers, where an effective increase in the sale and consumption of organic products is sought through the generation of awareness in the health care of the population [62].

## 3. Methodological Aspects

This research utilized the deductive method and contemplates general aspects to reach the specifics. It is of a descriptive–correlational type, because it is a representation of what happens to the behaviour and attitude of the consumer towards organic food products; on the other hand, it is correlational, because it seeks to associate and correlate the variables in addition to identifying their degree of determination. The primary source of data collection was a survey of 195 people, a sample that represents an economically active population of 76,113 individual consumers in Riobamba. Riobamba is considered a city, being the capital of the province of Chimborazo, and it concentrates an important population in relation to the rural communities. Different associations and producers of organic food products have this city as their main market, and thus they also have market coverage to sell their products. 

The study was carried out in 2020, in the period of the pandemic (Covid-19). The size of the sample (195 individuals) reflects a representative group of the population made up of different social classes and areas in which they live. All individuals in the sampling frame had the same probability of being chosen, and a study focused on a certain market segment is ruled out. The sample is determined based on the approach of Bencardino [73], considering a probability for or against 50%, a confidence level of 0.94, and a margin of error of 0.06, whose formula proposed by the author is:

$$n=\frac{z^{2}p\left(1-p\right)N}{e^{2}N+z^{2}p\left(1-p\right)}$$

$$n=\frac{{\left(1.7\right)}^{2}\;0.5\left(1-0.5\right)76113}{{\left(0.06\right)}^{2}\;\left(76,113\right)+{\left(1.96\right)}^{2}\left(0.5\right)\left(1-0.5\right)}\;\;n=195$$

The survey was conducted randomly until the sample size was completed. The proposed variables were developed from the literature in other fields, with very few uses in reference to the purchase of organic food products. Demographic and cultural variables are included as factors that determine consumer behaviour in terms of the purchase of organic products, and on the other hand, in terms of the motivation, emotions, and feelings related to the consumer’s attitude, they are constructed from Likert scales and are ordinal variables.

Another source of information was the consultation of documents related to the associations of producers of organic products and specialized literature in the field of organic food products production and marketing. We analysed data using the SPSS version 20 (IBM, New York, NY, USA).

## 4. Results

The survey applied to the economically active population of the city of Riobamba. The consulted population is made up of 55.3% women and 44.7% men with the age range between 18 and 30 years consist the majority (61.3%). The most prominent educational level was the higher level, with 56.3%, followed by the secondary level, with 27.4%; the social classes with the highest repetition are the middle class and the middle-low class with 63.7% and 19.5%, respectively. When purchasing a particular type of product or service, 82.1% of the population prefers to invest in food and health; when making purchases, 41.6% do not consult with anyone to do so, 28.4% consult with their partner or family, and 23.2% consult with their parents. In terms of the frequency of food purchases, 40.5% do so fortnightly, 35.1% do so weekly, and 13.7% do so two to three times a week.

On the other hand, interest in the consumption of organic products highlights that 47.9% are very interested in acquiring organic food products, and in fact, they are also frequent consumers and consider the purchase of organic foods to be linked to good health; 24.2% indicate that they are infrequent consumers and that they may purchase the product again. The attitude of consumers towards these products is mostly good, with a percentage of 52.4%.

The following cross tables show the interaction between the age variable and the influence of the attitude and behaviour of the consumers when purchasing organic products, Table 1.

Regarding the influence of consumer behaviour and attitude toward organic products, the survey showed that among the individuals aged between 18 and 30 years old, 31.6% consider that consumer behavior influences the consumer at the time of purchase, 33.3% indicate that attitude influences, and both behavior and attitude according to 35%. It follows that the purchase of organic products depends largely on attitude, followed by behavior, and that attitude is associated with motivation and emotion. Association between age and the variables which influence the purchase of organic products of the consumers was found statistically significant on Chi-squared test (*p* = 0.047). Table 2 shows the relationship between social class and interest in consuming products.

With regard to social class and interest in buying organic products, the most representative statistic is that, for the middle class, 55.4% are interested, 20.7% are very interested, and 16.2% are uninterested to consume organic products. Majority of the participants showed some degree of interest in consuming these products, due to their quality, price, and that they are linked to health. However, the association between social class and interest in organic product consumption was not found statistically significant on Chi-squared test (*p* = 0.10).

Linear regression is used to test the hypotheses. Being:

**Hypothesis** **H1.** *The cultural and demographic aspects are the most important factors that determine the behavior of the consumer in the purchase of organic products in the city of Riobamba, the model is represented mathematically as follows*:

Y_(t)_ = β_0_ + β_1_X_1_ + β_2_X_2_ + β_3_X_3_ + ε

where:

Y(t): dependent variable (consumer buying behavior)

Y(t): consumer buying behavior (CBB)

β_0_: constant.

β: measures variables behaviour similarity

X_1_ + X_2_ + X_3_: independent variables

X_1_= demographics gender (DG)

X_2_= demographic social status (DSS)

X_3_= cultural factors (CF)

Y (CBB) = 1.378 + 0.062 (DG) + 0.041 (DSS) + 0.084 (CF) + ε

The model establishes a value of the R correlation of 27.1%; that is to say, there is a symbolic but not representative relationship between the dependent and independent variables. The degree of dependency is also very low, reaching 7.4%. This means that the culture and demographic variables explain 27.1% of consumer behavior in the process of purchasing organic products. It should be noted that there are other variables that could be explanatory which were not addressed in the present investigation. Both variables have been considered, because in the literature and the a priori conclusions made in conversations, academic talks, and seminars, mention is often made that culture and demographics would be the main variables of consumer behaviour at the time of purchase of organic products in the city of Riobamba. Therefore, the proposed hypothesis is accepted, but it is not significant in the values found.

**Hypothesis** **H2.** *Emotions, feelings and motivation are the factors that are significantly related to the consumer’s attitude towards the purchase of organic products in the city of Riobamba.*

Where:

Y(t): dependent variable (consumer attitude towards purchase)

Y(t): dependent variable (CAP)

β_0_: constant.

β: measures variables behaviour similarity

X_1_ + X_2_ + X_3_: independent variables

X_1_= Emotions (E)

X_2_= Feeling (F)

X_3_= Motivation (M)

Y (CAP) = 2.136 + 0.582 (E) + 0.491 (F) + 0.411(M) + ε

Model summary determined a correlation of 72.4% and a determination coefficient of 48%, in addition to an ANOVA (sigma of 0.000); based on these values, it is established that emotions, feelings, and motivation explain 72.4% of the model and proposed hypothesis, and given that the value of sigma is 0.000, the proposed alternative hypothesis is accepted. These are, therefore, the most significant variables in the consumer’s attitude towards the purchase of organic products. In response to the question about the psychological factors that are necessary to take into account during the process of buying organic products, the respondents affirmed in a significant percentage that emotions, feelings, and motivation are very necessary (43.5%) or necessary (34.7%) on a Likert scale. This affirms and sustains the significance of the presented model.

The way in which culture and demographic factors influence consumer behavior is not decisive, so the purchase of organic products is not necessarily determined by behavior; meanwhile, the variables of emotions, feelings, and motivation are related to the attitude of the consumer, and determine in an important way the purchase of organic products. This situation helps us to understand that in the context of Ecuador, the attitudinal issue has a greater degree of importance in relation to consumer behavior in the purchase of organic products. It should also be noted that the purchasing preferences for organic products are concentrated on quality, ease of logistics/access, and prices.

## 5. Discussion

Understanding consumer behaviours and attitudes is one of the most-mentioned topics today, with the objective of knowing the internal and external factors that influence consumers’ purchase decisions. Consumers, thanks to these factors, may have individual or family-level behaviours or attitudes, that is, sometimes decisions are made under the influence of other people or it may simply be the combination of the two parties at the time of purchase. Therefore, the understanding of consumer needs and desires which influence their purchase behaviours [65] is an area in which marketing has concentrated great efforts. In addition, the study of factors that intervene in consumer behaviour, such as cultural, demographic, psychological, and social factors, is also a substantial focus, with the aim of understanding and anticipating consumer behaviour [70].

There are many people who believe that purchasing decisions are based on rational analysis, but few know that this is far from reality, as it is the emotions that influence the moment of buying. One study reveals that those advertisements that have emotional content have a greater impact on the consumer, which influences their purchase [48]; but often, organizations are only interested in selling their products without taking into account the emotional aspects of their buyers [57]. There are few who know that it is necessary for there to be emotional content in a brand or mental representation of the product, so that the consumer decides his purchase and becomes a potential consumer. Thus, motivation in consumers is expressed in different ways, being the internal aspect which drives action that arises due to a need that might be satisfied or unsatisfied; that is, it can be positive, reaching expectations, or negative, avoiding fears. 

Consumer purchase motivation is a multidimensional construct with cognitive, affective, and moral elements. In the context of motivation linked to attitude about and purchase of organic products, literature is scarce; however, there are important contributions that establish that the purchase motivation of agricultural products influences the attitude, and this determines the purchase and consumption of products [62]. In this line, they also agree that motivation is the main decision-maker that has an effect on the consumer’s attitude towards access to essential products, including agricultural, livestock, and meat products. Therefore, the affirmation of this relationship in the field of organic products is also established in these terms.

The sentiment of consumers’ is directly related to emotions. Sentiment analysiswhich is the process of uncovering emotions in online communications to find out how people feel about your product. Sentiment analysis therefore consists of evaluating emotions and opinions. Studies reveal that sentiment analysis of emotions uses advanced artificial intelligence technologies such as natural language processing, text analytics, and data science to identify, extract, and study subjective information. In simpler terms, to classify a text as positive, negative, or neutral. Traditional metrics like number of views, likes, shares, comments, etc., focus on quantity, and sentiment analysis goes beyond numbers and focuses on the quality of interactions between the public and the organization [39]. In the field of organic product purchasing processes, the literature is limited, and some works indicate that feelings are related to the origin of the product, while others indicate that they are related to the way in which they are exposed at the point of sale, highlighting a relationship with emotions and attitude. In this context, the results of the present investigation coincide with this approach; that is, feelings have a significant relationship in the attitude of the consumers of organic products.

In the case of the variables of culture and demographics linked to consumer behaviour when making a purchase, they are broad [55]. They also maintain that there is a relationship between these variables, including perceived quality, psychological processes, expectations, and loyalty. Research carried out in the field of agricultural products refers to the appearance, culture, demographic, psychological, and sociological aspects that would be linked mainly to consumer behaviour [49,58]. As mentioned above, consumer preference for organic products is also influenced by quality [74,75]. Price is another important factor that consumers consider when buying organic products [76]. Likewise, it is observed that the attitude towards organic food is the main determinant of the purchase intention, and together with health concerns and the perception of quality, it has a significant impact on the access and consumption of organic products [77]. Therefore, this research exposes the factors that are linked to the behaviour and attitude of the consumer in the purchase of organic products; as evidenced in the case of culture and demographics, there is a non-significant relationship with behaviour [78], while within the attitude of the consumer during the purchase, elements such as motivation, feelings, and emotions are identified. In relation to other presented research works, it seems important to also pay attention to other variables such as the price and quality of the products.

## 6. Conclusions

This research reveals that emotions, feelings, and motivation are the factors that are significantly related to the attitude of the consumers toward the purchase of organic food products in the city of Riobamba. The literature corroborates this statement, and its contribution to the study of these constructs within the field of consumers’ attitudes toward organic products also becomes a focus for future research. In the case of culture and demographic variables, they do not significantly determine consumer behaviour, contrary to the theoretical approach of products and services in another field, different from ecological products. This conclusion offers a position regarding the attitude and behaviour of the consumer towards the purchase of organic products in an urban context, where the consumption of these products is not massive, it is in a growing phase and promoted by associations and organizations that produce and market products, with traditional marketing systems and a phase of promoting them, which could also influence the results obtained. This investigation is contributing to an underdeveloped field, and, the proposed variables have been developed by the literature in other disciplines with very few applications in the organic field, which represents the importance of this study. One limitation of this paper is the fact that psychological and sociological variables were not included, which may be variables analyzed in future research works, which a priori, could also explain the attitude of the consumer in the purchase of organic products.

The research presents some limitations, and they have to do with the approach to other variables linked to the behaviour and attitudes of consumers of organic products, namely price, quality, logistic means, and market. However, these elements can be addressed in a second investigation, this will allow for an overview and a complete reading of the study phenomenon in the developed context. It is also recommended that public policies be designed and strategies be aimed at the production and promotion of consumption of organic products in such a way, that the consumer has more information about the characteristics and benefits of organic products, as this will help create better attitudes and behaviours in the purchase decisions of consumers, and consequently, in the care and improvement of the health of the population.

## Figures and Tables

**Table 1:** Association between age and consumer behaviour and attitude.

		Variables Influence the Purchase of Organic Products			
		Behaviour	Attitude	Behaviour and Attitude	Total
Age	18 to 30 years	37	39	41	117
		31.6%	33.3%	35.0%	100%
	31 to 43 years	12	10	22	44
		27.3%	22.7%	50.0%	100%
	44 to 56 years	3	4	11	18
		16.7%	22.2%	61.1%	100%
	57 to 69 years	1	0	8	9
		11.1%	0%	88.9%	100%
	70 years or more	1	0	1	2
		50.0%	0%	50.0%	100%
Total		54	53	83	190
		28.4%	27.9%	43.7%	100%

**Table 2:** Association between social class and interest in consuming organic products.

Would You Be Interested in Consuming Organic Products?							
		Very Interested	Interested	Neutral	Uninterested	Very Uninterested	Total
Social class	Low	4	3	1	1	0	9
		44.4%	33.3%	11.1%	11.1%	0%	100.0%
	Middle-low	12	10	9	6	0	37
		32.4%	27.0%	24.3%	16.2%	0%	100.0%
	Middle	25	67	21	7	1	121
		20.7%	55.4%	17.4%	5.8%	0.8%	100.0%
	Middle-High	2	8	5	0	1	16
		12.5%	50.0%	31.3%	0%	6.3%	100.0%
	High	3	3	1	0	0	7
		42.9%	42.9%	14.3%	0%	0%	100.0%
Total		46	91	37	14	2	190
		24.2%	47.9%	19.5%	7.4%	1.1%	100%

## Data Availability

Data set used for this study is available from the corresponding author on reasonable request.

## References

[1] Asiegbu I., Daubry M., Iruka C. (2012). Consumer Attitude: Some Reflections on Its Concept, Trilogy, Relationship with Consumer Behavior, and Marketing Implications. Eur. J. Bus. Manag..

[2] Ajzen I. (2015). Consumer Attitudes and Behavior. Handbook of Consumer Psychology.

[3] Kostadinova E. (2016). Sustainable Consumer Behavior: Literature Overview. Econ. Altern..

[4] Gajjar N. (2013). Factors Affecting Consumer Behavior. Rev. Index. Mon. J..

[5] Vinerean S., Cetina I., Dumitrescu L., Tichindelean M. (2013). The Effects of Social Media Marketing on Online Consumer Behavior. Int. J. Bus. Manag..

[6] Haytko D., Matulich E. (2008). Green advertising and environmentally responsible consumer behaviors: Linkages examined. J. Manag. Mark. Res..

[7] Laoviwat P., Suppapanya P., Yousapronpaiboon K. (2014). A Study of Demographics Influencing on Consumer Behavior and Attitude towards Brand Equity of Optical Business in Thailand. Int. J. Trade Econ. Financ..

[8] Kim J., Lee M., Han H. (2020). Smart hotels and sustainable consumer behavior: Testing the effect of perceived performance, attitude, and technology readiness on word-of-mouth. Int. J. Environ. Res. Public Health.

[9] Smith J., Terry D., Louis W., Kotterman D. (2008). The Attitude–Behavior Relationship in Consumer Conduct: The Role of Norms, Past Behavior, and Self-Identity. J. Soc. Psychol..

[10] Argyriou E., Melewar T. (2011). Consumer attitudes revisited: A Review of attitude theory in marketing research. Int. J. Manag. Rev..

[11] Gemina D., Andari T., Kusuma I. (2013). Consumer Behavior on The Choice of Typical Regional Food Products Based on External and Internal Factors, Perception, Attitude and Consumer Preference. Int. J. Adv. Sci. Eng. Inf. Technol..

[12] Adhitiya L., Astuti R. (2019). The Effect of Consumer Value on Attitude Toward Green Product and Green Consumer Behavior in Organic Food. IPTEK J. Proc. Ser..

[13] Dhaliwal A., Singh D.P., Paul J. (2020). The consumer behavior of luxury goods: A review and research agenda. J. Strategy Mark..

[14] Rana J., Paul J. (2017). Consumer behavior and purchase intention for organic food: A review and research agenda. J. Retail. Consum. Serv..

[15] Lazaroiu G., Andronie M., Uţă C., Hurloiu I. (2019). Trust Management in Organic Agriculture: Sustainable Consumption Behavior, Environmentally Conscious Purchase Intention, and Healthy Food Choices. Front. Public Health.

[16] Araújo H., Marjotta-Maistro M. (2022). Profiling the consumer of agroecological products using cluster analysis. Rev. Econ. Sociol. Rural.

[17] Charrua A., Havik P., Bandeira S., Catarino L., Ribeiro-Barros A., Cabral P., Moldão M., Romeiras M. (2021). Food security and nutrition in mozambique: Comparative study with bean species commercialised in informal markets. Sustainability.

[18] Park J., Kim S., Kim S., Jeoung E., Park J. (2020). Household food insecurity: Comparison between families with and without members with disabilities. Int. J. Environ. Res. Public Health.

[19] Little M., Sylvester O. (2022). Agroecological producers shortening food chains during COVID-19: Opportunities and challenges in Costa Rica. Agric. Hum. Values.

[20] Tittarelli F., Saba A., Di Pierro M., Ciaccia C. (2022). Food Citizenship as an Agroecological Tool for Food System Re-Design. Sustainability.

[21] Mehrabi S., Perez-Mesa J., Giagnocavo C. (2022). The Role of Consumer-Citizens and Connectedness to Nature in the Sustainable Transition to Agroecological Food Systems: The Mediation of Innovative Business Models and a Multi-Level Perspective. Agriculture.

[22] Kiliç I., Bozkurt Z. (2020). Assessment of Turkish consumer attitudes using an animal welfare attitude scale (AWAS). Vet. Mex..

[23] Schiller K., Godek W., Klerkx L., Poortvliet P. (2020). Nicaragua’s agroecological transition: Transformation or reconfiguration of the agri-food regime?. Agroecol. Sustain. Food Syst..

[24] González F., León L., López-Estébanez L. (2022). Family Farming as a Key Element of the Multifunctional and Territorialized Agrifood Systems as Witnessed in the South Pacific Region of Costa Rica. Land.

[25] Muñoz E., Niederle P., de Gennaro C., Roselli L. (2021). Agri-food markets towards agroecology: Tensions and compromises faced by small-scale farmers in Brazil and Chile. Sustainability.

[26] Coca E. (2021). Food Procurement in Post-neoliberal Countries: Examples from South America. Agrar. South.

[27] Gallegos-Riofrío C., Waters W., Carrasco-Torrontegui A., Iannotti L. (2021). Ecological community: Heterarchical organization in a contemporary agri-food system in Northern Andes. Geoforum.

[28] Moreno-Miranda C., Paredes M., Solís N., Moreno R., Rama D. (2020). Structural analysis of nontraditional Andean fruit chains: The case of the Inca berry agri-food network in Ecuador. J. Agric. Environ. Int. Dev..

[29] Vallejo-Rojas V. (2016). Active Transformative Pathways for Local Agri-Food Systems: Drawing and Applying an Integrated Framework to Assess Agri-Food Systems Vulnerability Under the Political Paradigm of Food Sovereignty in Ecuadorian Andes. Ph.D. Dissertation.

[30] Ayuda M., Belloc I., Pinilla V. (2022). Latin American Agri-Food Exports, 1994–2019: A Gravity Model Approach. Mathematics.

[31] Intriago R., Amézcua R.G., Bravo E., O’Connell C. (2017). Agroecology in Ecuador: Historical processes, achievements, and challenges. Agroecol. Sustain. Food Syst..

[32] Sherwood S., Arce A., Paredes M. (2018). Affective Labor’s ‘unruly edge’: The pagus of Carcelen’s Solidarity & Agroecology Fair in Ecuador. J. Rural Stud..

[33] Deaconu A., Berti P.R., Cole D., Mercille G., Batal M. (2021). Agroecology and nutritional health: A comparison of agroecological farmers and their neighbors in the Ecuadorian highlands. Food Policy.

[34] Dos Santos P., Bevilacqua P. (2019). Family farming in agroecological transition: A look at the marketing of milk and dairy products in municipalities of the Zona da Mata of Minas Gerais State, Brazil. Cienc. Rural.

[35] Brune S., Knollenberg W., Stevenson K., Barbieri C., Schroeder-Moreno M. (2021). The Influence of Agritourism Experiences on Consumer Behavior toward Local Food. J. Travel Res..

[36] Babin N. (2015). The Coffee Crisis, Fair Trade, and Agroecological Transformation: Impacts on Land-Use Change in Costa Rica. Agroecol. Sustain. Food Syst..

[37] Parodi G. (2018). Agroecological transition and reconfiguration of horticultural work among family farmers in Buenos Aires, Argentina. Cah. Agric..

[38] Deaconu A. (2021). Promoting traditional foods for human and environmental health: Lessons from agroecology and Indigenous communities in Ecuador. BMC Nutr..

[39] Thompson J., Scoones I. (2009). Addressing the dynamics of agri-food systems: An emerging agenda for social science research. Environ. Sci. Policy.

[40] Just F., Emmanuel A., Oteng-Seifah S., Theophilus A.-K., Theophilus E.-A., Cimpoies D., Zhemoyda O.V., Portyanko M., Danilowska A., Danilowska A. (2007). Sustainable Rural Development: What Is the Role of the Agri-Food Sector?.

[41] Otiman P., Toderoiu F., Alexandri C., Florian V., Gavrilescu C., Ionel I., Sima E., Tudor M.M. (2014). Sustainable Development Strategy for the Agri-food Sector and Rural Area—Horizon 2030. Procedia Econ. Financ..

[42] Deaconu A., Mercille G., Batal M. (2019). The Agroecological Farmer’s Pathways from Agriculture to Nutrition: A Practice-Based Case from Ecuador’s Highlands. Ecol. Food Nutr..

[43] Peighambari K., Sattari S., Kordestani A., Oghazi P. (2016). Consumer Behavior Research: A Synthesis of the Recent Literature. SAGE Open.

[44] Chovanová H., Korshunov A., Babčanová D. (2015). Impact of Brand on Consumer Behavior. Procedia Econ. Financ..

[45] Cachon G., Swinney R. (2011). The value of fast fashion: Quick response, enhanced design, and strategic consumer behavior. Manag. Sci..

[46] Yuan C., Wang S., Yu X. (2020). The impact of food traceability system on consumer perceived value and purchase intention in China. Ind. Manag. Data Syst..

[47] Zhong Y., Moon H.C. (2020). What Drives Customer Satisfaction, Loyalty, and Happiness in Fast-Food Restaurants in China? Perceived Price, Service Quality, Food Quality, Physical Environment Quality, and the Moderating Role of Gender. Foods.

[48] Cheah I., Shimul A., Liang J., Phau I. (2022). Consumer attitude and intention toward ridesharing. J. Strategy Mark..

[49] Dhaoui O., Nikolaou K., Mattas K., Baourakis G. (2020). Consumers’ attitude towards alternative distribution channels of fresh fruits and vegetables in Crete. Br. Food J..

[50] Liu Q., Zhang X., Huang S., Zhang L., Zhao Y. (2020). Exploring consumers’ buying behavior in a large online Promotion Activity: The role of Psychological Distance and Involvement. J. Theor. Appl. Electron. Commer. Res..

[51] Le N., Hoang T. (2020). Measuring Trusts and the Effects on The Consumers’ Buying Behavior. J. Distrib. Sci..

[52] Alhamdi F. (2020). Role of packaging in consumer buying behavior. Manag. Sci. Lett..

[53] Nosi C., Zollo L., Rialti R., Ciappei C. (2020). Sustainable consumption in organic food buying behavior: The case of quinoa. Br. Food J..

[54] Ittaqullah N., Madjid R., Suleman N. (2020). The effects of mobile marketing, discount, and lifestyle on consumers’ impulse buying behavior in online marketplace. Int. J. Sci. Technol. Res..

[55] Burton J., Gollins J., McNeely L., Walls D. (2019). Revisiting the relationship between ad frequency and purchase intentions how affect and cognition mediate outcomes at different levels of advertising frequency. J. Advert. Res..

[56] Sheikh Q. (2019). Consumer Buying Decision Process toward products. Int. J. Sci. Res. Eng. Dev..

[57] Woo E., Kim Y. (2019). Consumer attitudes and buying behavior for green food products: From the aspect of green perceived value (GPV). Br. Food J..

[58] Lee T., Fu C., Chen Y. (2020). Trust factors for organic foods: Consumer buying behavior. Br. Food J..

[59] Li Y., Wang H., Zhang P., Popkin B.M., Coyle D.H., Ding J., Dong L., Zhang J., Du W., Pettigrew S. (2022). Nutritional Quality of Pre-Packaged Foods in China under Various Nutrient Profile Models. Nutrients.

[60] Chaparro-Africano A., Garzón-Méndez J. (2021). Consumer profile and factors determining the purchase of agroecological products. A case study: UNIMINUTO Agroecological Fair and Minuto de Dios Solidarity Market, Colombia. Agron. Colomb..

[61] Baydas A., Yalman F., Bayat M. (2021). Consumer attitude towards organic food: Determinants of helathy behavior. Mark. Manag. Innov..

[62] Radulescu V., Cetina I., Cruceru A., Goldbach D. (2021). Consumers’ attitude and intention towards organic fruits and vegetables: Empirical study on romanian consumers. Sustainability.

[63] Saxena M., Sharma M., Jain A. (2021). Impact of food selection and usage pattern on consumers’ attitude towards food label information. J. Postharvest Technol..

[64] Tandijaya T., Hatane S. (2021). Viral Marketing Message, Consumers’ Attitude towards Viral Marketing, Competitiveness Ability, and Business Performance. J. Manaj. Pemasar..

[65] Wang M., Kumar V., Ruan X., Saad M., Garza-Reyes J., Kumar A. (2021). Sustainability concerns on consumers’ attitude towards short food supply chains: An empirical investigation. Oper. Manag. Res..

[66] Yeh C.-H., Menozzi D., Török Á. (2020). Eliciting Egg Consumer Preferences for Organic Labels and Omega 3 Claims in Italy and Hungary. Foods.

[67] Cordero-Ahiman O.V., Vanegas J.L., Fernández-Lucero C.A., Torres-Torres D.F., Ayaviri-Nina V.D., Quispe-Fernández G.M. (2022). Responsible Marketing in the Traffic Light Labeling of Food Products in Ecuador: Perceptions of Cuenca Consumers. Sustainability.

[68] Roseira C., Teixeira S., Barbosa B., Macedo R. (2022). How Collectivism Affects Organic Food Purchase Intention and Behavior: A Study with Norwegian and Portuguese Young Consumers. Sustainability.

[69] Melovic B., Cirovic D., Dudic B., Vulic T.B., Gregus M. (2020). The Analysis of Marketing Factors Influencing Consumers’ Preferences and Acceptance of Organic Food Products—Recommendations for the Optimization of the Offer in a Developing Market. Foods.

[70] Leyva-Hernández S.N., Toledo-López A., Hernández-Lara A.B. (2021). Purchase Intention for Organic Food Products in Mexico: The Mediation of Consumer Desire. Foods.

[71] Bosona T., Gebresenbet G. (2018). Swedish Consumers’ Perception of Food Quality and Sustainability in Relation to Organic Food Production. Foods.

[72] Zheng G.-W., Akter N., Siddik A.B., Masukujjaman M. (2021). Organic Foods Purchase Behavior among Generation Y of Bangladesh: The Moderation Effect of Trust and Price Consciousness. Foods.

[73] Bencardino C.M. (2012). Estadística y Muestreo.

[74] Rojík S., Zámková M., Chalupová M., Pilař L., Prokop M., Stolín R., Malec K., Appiah-Kubi S.N.K., Maitah M., Dziekánski P. (2022). Pre-COVID-19 Organic Market in the European Union—Focus on the Czech, German, and Slovak Markets. Agriculture.

[75] Guiné R.P.F., Florença S.G., Costa D.T.V.A., Çelik S., Ferreira M., Cardoso A.P., Çetin S., Costa C.A. (2022). Comparative Study about the Consumption of Organic Food Products on Samples of Portuguese and Turkish Consumers under the COVID-19 Pandemic Context. Agronomy.

[76] Smiglak-Krajewska M., Wojciechowska-Solis J. (2021). Consumer versus Organic Products in the COVID-19 Pandemic: Opportunities and Barriers to Market Development. Energies.

[77] Vega-Muñoz A., Gil-Marín M., Contreras-Barraza N., Salazar-Sepúlveda G., Losada A.V. (2022). How to Measure Organic Fruit Consumer Behavior: A Systematic Review. Horticulturae.

[78] Teixeira S.F., Barbosa B., Cunha H., Oliveira Z. (2022). Exploring the Antecedents of Organic Food Purchase Intention: An Extension of the Theory of Planned Behavior. Sustainability.

